# Simulative Global Warming Negatively Affects Cotton Fiber Length through Shortening Fiber Rapid Elongation Duration

**DOI:** 10.1038/s41598-017-09545-y

**Published:** 2017-08-23

**Authors:** Yanjiao Dai, Jiashuo Yang, Wei Hu, Rizwan Zahoor, Binglin Chen, Wenqing Zhao, Yali Meng, Zhiguo Zhou

**Affiliations:** Key Laboratory of Crop Physiology & Ecology, Ministry of Agriculture, College of Agriculture, Nanjing Agricultural University, Nanjing, Jiangsu Province China

## Abstract

Global warming could possibly increase the air temperature by 1.8–4.0 °C in the coming decade. Cotton fiber is an essential raw material for the textile industry. Fiber length, which was found negatively related to the excessively high temperature, determines yarn quality to a great extent. To investigate the effects of global warming on cotton fiber length and its mechaism, cottons grown in artificially elevated temperature (34.6/30.5 °C, T_day_/T_night_) and ambient temperature (31.6/27.3 °C) regions have been investigated. Becaused of the high sensitivities of enzymes V-ATPase, PEPC, and genes *GhXTH1* and *GhXTH2* during fiber elongation when responding to high temperature stress, the fiber rapid elongation duration (FRED) has been shortened, which led to a significant suppression on final fiber length. Through comprehensive analysis, T_night_ had a great influence on fiber elongation, which means T_n_ could be deemed as an ideal index for forecasting the degree of high temperature stress would happen to cotton fiber property in future. Therefore, we speculate the global warming would bring unfavorable effects on cotton fiber length, which needs to take actions in advance for minimizing the loss in cotton production.

## Introduction

The mean air temperature surrounding us would increase by 1.8 to 4.0 °C in the coming decades, which is unfavorable to agricultural production^1^. In the last five decades, there were over 20 days per year with the maximum daily temperature over 38 to 40 °C in the cotton belt of China^2, 3^, and high temperature events would happen more frequently as a result of global warming. High temperature stress had strong negative correlations with cotton (*Gossypium hirsutum* L.) lint yield^4^ and fiber qualities, especially fiber length^5^. Ramey and Jr.(1986) defined the optimal temperature range of 15 to 21 °C for cotton fiber development^6^, otherwise disadvantageous temperature would lead to poor cotton fibers^7–10^.

Getting longer cotton fibers has always been our goal since it determines yarn quality a lot. Existing knowledge shows, the final fiber length mostly depends on its elongation rate and the duration of rapid elongation process^11, 12^. High speed and persistent elongation are basic features required for long fibers^11^. However, incongruous temperature during fiber growth limits this potential^13^. It has been proved that the temperatures during fiber initiation and early elongation stages had profound influences on fiber development^14^. Gipson and Ray (1969) further indicated that the fiber elongation rate was highly sensitive to the temperature before 15 days post anthesis (DPA)^15^.

Cell turgor, the main driving force for cotton fiber elongation^16^, was generated by the accumulation of osmotically active solutes^17^, which mainly includes malate, K^+^ and soluble sugars^18^. The enzymes related to osmoregulation and carbohydrate metabolism^19, 20^ are critical for fiber elongation. Vacuolar ATPase (V-ATPase, EC 3.6.1.3) is responsible for most ionic transportation, including K^+^, by stimulating the secondary active transportation across tonoplast^21^. *GhPEPC1* and *GhPEPC2* are main regulatory genes for phosphoenolpyruvate carboxylase (PEPC, EC 4.1.1.31) synthesis in cotton fiber^18^, which could catalyse malate synthesis inside the cytoplasm, afterwards^22^. Sucrose synthase (SuSy, EC 2.4.1.13) and invertase (INV, EC 3.2.1.26) are the two key enzymes responsible for sucrose cleavage into hexoses^23^, the main existing form of soluble sugar in cotton fiber^20^. SuSy activity has been additionally verified crucial for promoting fiber elongation, and could even affect cotton fiber yield^23^. The reduction of vacuolar INV (VINV) activity has an apparent suppression on fiber initiation, which could result in fiberless cotton seed^24^.

Fiber length is also limited by the cell wall relaxation process^25, 26^, which is also related to some enzymes expression and activities. These enzymes have been viewed as catalysts, which weaken the cell wall for permitting the turgor-driven extension. *Expansin* is one of the endogenous regulators for plant cell enlargement^27^. The expression level of *Expansin* remained high during the early stage of elongation (6 to 8 DPA) and decreased rapidly afterwards^16^. *GhXTH1*, the key gene regulating xyloglucan endotransglycosylase/hydrolase (XTH) synthesis, is another key regulator on cell wall loosening^26^. Highly-expressed *GhXTH1* enhanced the XTH activity and resulted in longer fibers^28^. Genetic improvements in fiber quality traits give competitive advantages to cotton production than ever before. However, adverse environmental conditions, such as global warming, shows strongly negative effect on fiber properties and might mask genetic advantages^4, 29^.

Cotton has various levels of temperature sensitivity^30^. Brown and Oosterhuis (2004) demonstrated the sensitivities of modern cultivars and obsolete cultivars to extreme temperature were different, but they could gain good yield and fiber properties under respective breeding years^31^. Here, we used two cotton cultivars namely Simian 3 and Siza 3 to perform the current study, which have similar ecological fitness but adapt to different ages. We aimed to explore an improved understanding of the thermo stabilities from these cotton cultivars, and characterize the responses of the key enzymes and genes related to cotton fiber elongation under artificially elevated temperature, for sustaining our result in physiological terms.

## Results

**Figure 1:**
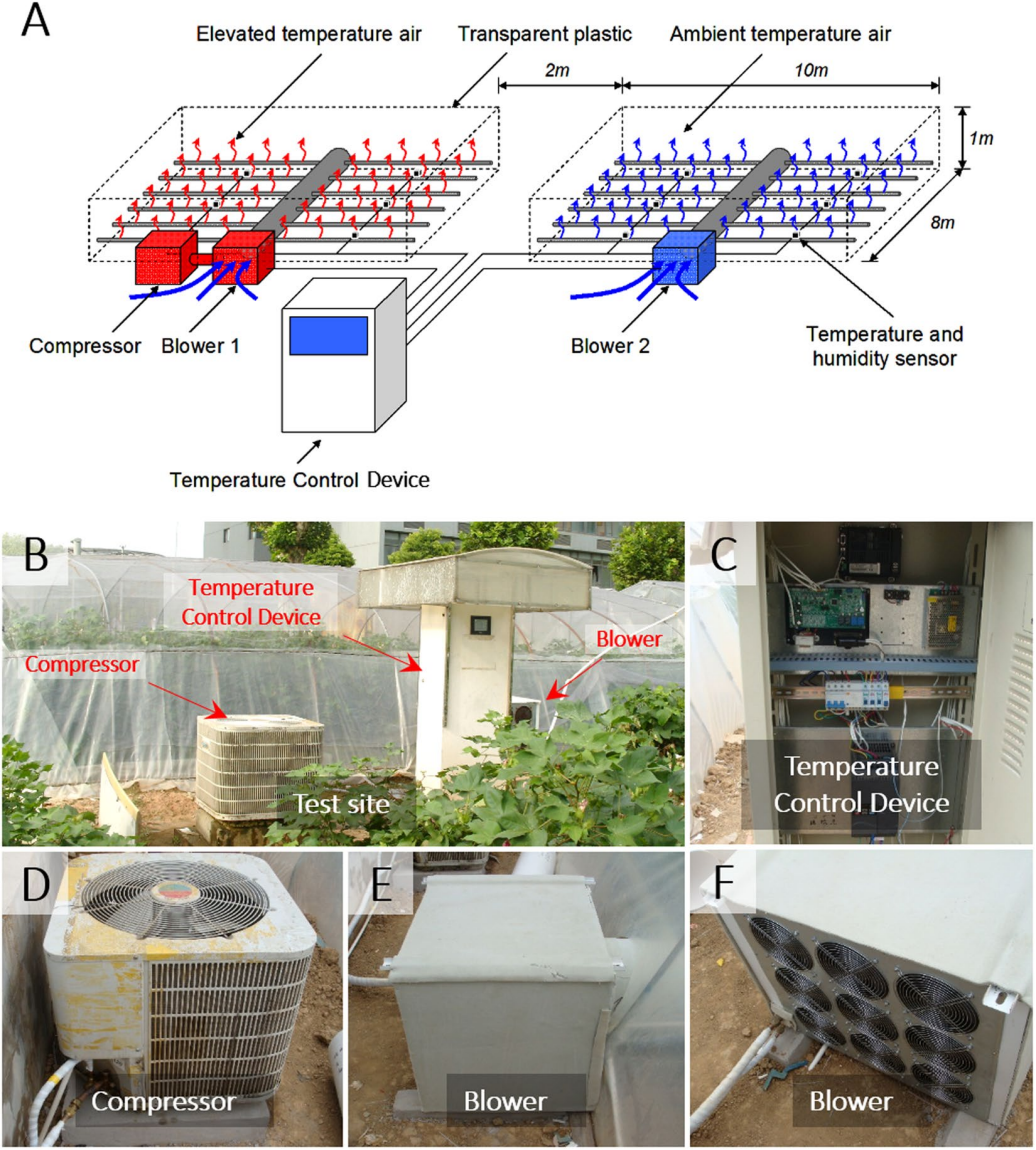
Temperature Control System. Structural diagram (**A**) and actual pictures (**B**–**F**) of temperature control device. The temperature elevation module works like an air conditioner, which contains the control module (**C**), compressor (**D**), and blower (**E**,**F**). Cotton field was surrounded by 1 m height transparent plastic, and covered by voile for preventing pests and hailstone.

**Figure 2:**
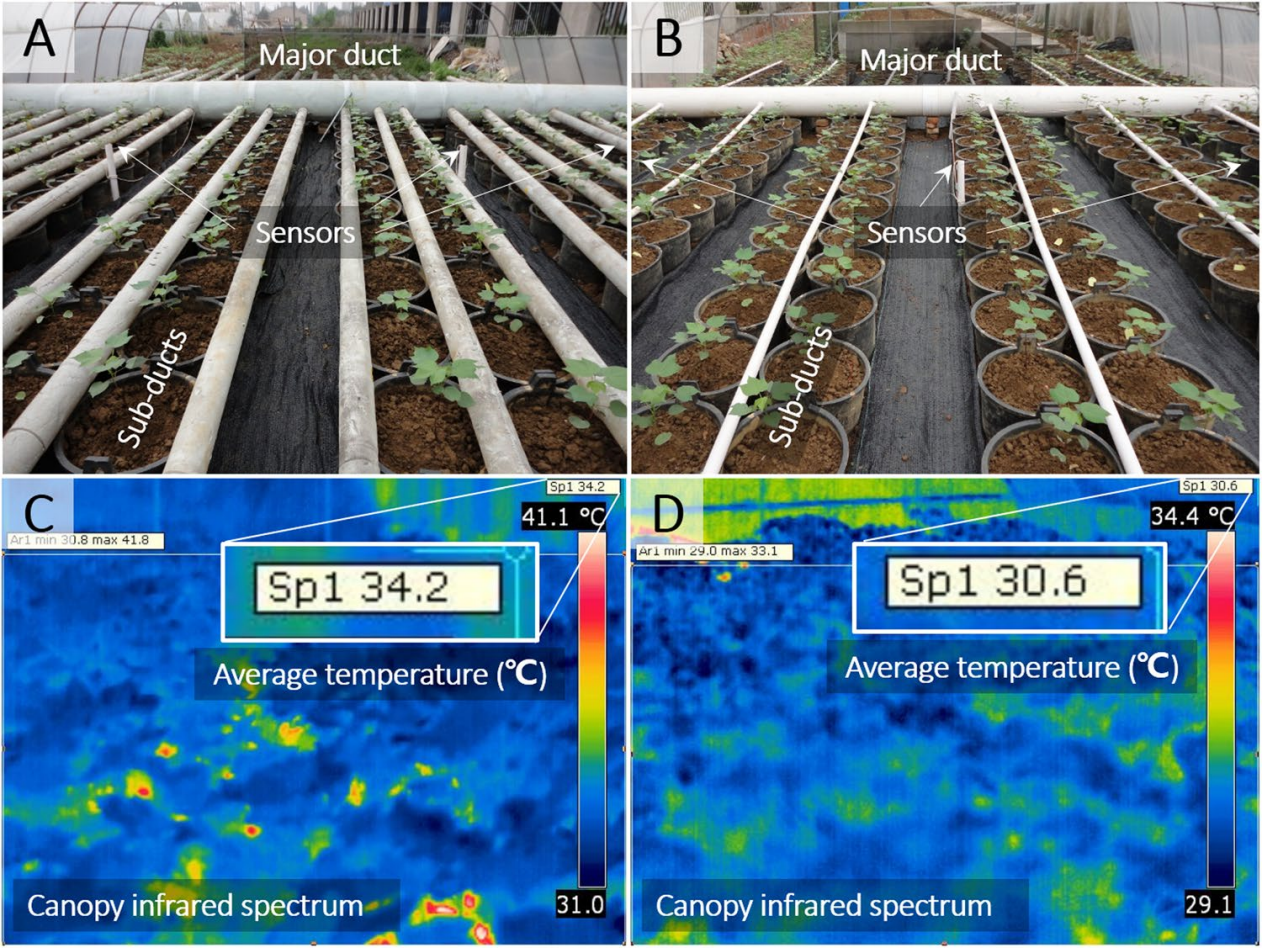
Ducts arrangement in cotton field, and the infrared spectrum of cotton canopy. In the Elevated temperature region (**A**), warm air had been blown into a major duct with 36 sub-ducts connected. The ducts is made of PVC, and covered with insulation sponge. In the control region (**B**), ambient (atmospheric) air had been blown into a major duct with 12 sub-ducts connected. Six temperature sensors were equidistributed in each region, which were located at the height of 6^th^ fruiting branch (about 80 cm off the ground). The canopy infrared spectrum of two temperature regions were presented (**C**,**D**), with a 3.6 °C difference in average between two regions.

**Figure 3:**
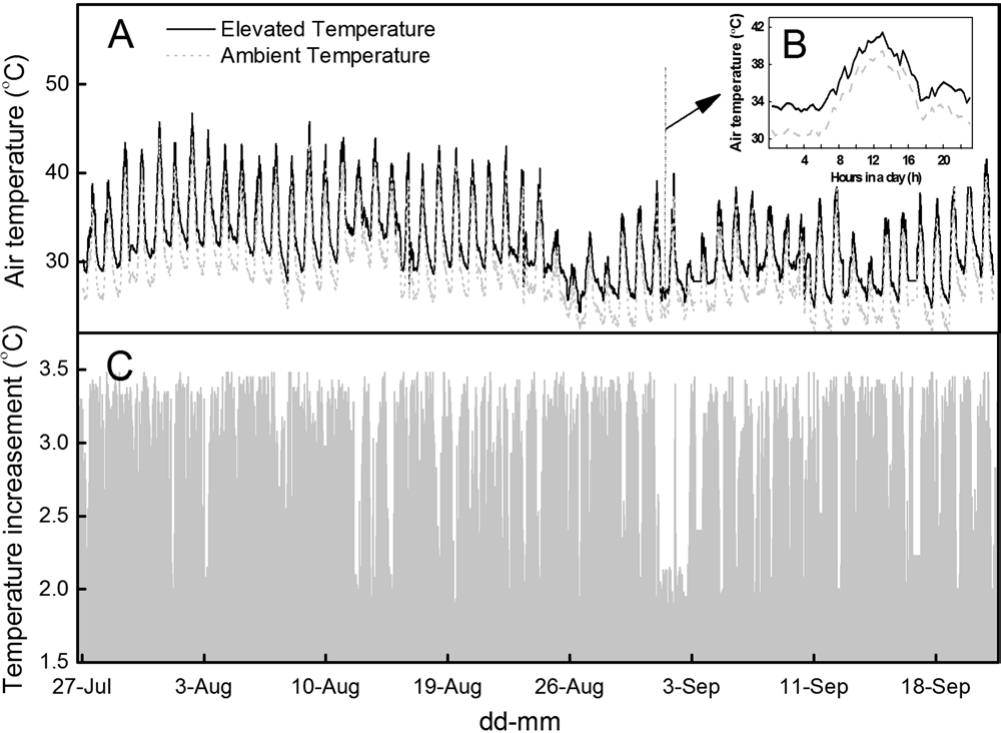
Air temperature recording. Air temperature in two regions during flowering season (Jul-27 to Sep-21, **A**), within 24 hours (**B**), and the temperature increment between two treatments during flowering season (**C**) in 2010. Temperature data were recorded at every 20 min by the temperature control device (**A**,**C**). A consistent air temperature increment by 2–3.5 °C existed all across fiber development (**C**). The mean daily air temperature (MDT) and other relative indices were calculated by the temperature control device.

A Temperature Control System (Figs 1 and 2) was built for creating a persistent air temperature elevation by 2–3.5 °C in cotton field, during cotton flowering season (Fig. 3). The mean daily temperature (MDT), mean daytime temperature (T_day_/T_d_), mean nighttime temperature (T_night_/T_n_), daily maximum temperature (T_max_), and daily minimum temperature (T_min_), during cotton fiber elongation were recorded (Table 1). The ambient MDT in 2011 (29.1 °C) was similar to 2012 (29.4 ± 0.5 °C), but lower than 2010 (31.6 ± 0.2 °C). However, as a result of temperature elevation, the MDT increment was relatively stable, which were 3.1 °C (mean of Simian 3 and Siza 3), 3.1 °C and 2.8 °C in 2010, 2011 and 2012, respectively. It was reported that the upper limit of the tolerable temperature for cotton fiber growth was 32/28 °C (T_d_/T_n_)^32, 33^. The T_d_/T_n_ during cotton fiber elongation in elevated temperature region was 36.5/31.6, 33.7/29.8 °C and 33.8/29.9 °C for Simian 3, while 36.6/31.7 °C, 33.7/30.0 °C and 33.3/29.8 °C for Siza 3, in 2010, 2011 and 2012, respectively (Table 1). This indicated that the temperature in elevation regions throughout the three years had exceeded the endurable region. Additionally, the temperature elevation had also prominently enhanced the daily hours that temperature >32 °C, which were 9–17 hours in elevated temperature region, but only 4–8 hours in control region (Table 1). It is worthy to mention that no significant difference between two temperature regions on their relative humidities has been found (Table 2).

**Table 1:** Temperature indices during cotton fiber elongation in two temperature regimes from 2010 to 2012.

Year	Cultivar	Temperature regime	MDT (°C)	T_d_ (°C)	T_n_ (°C)	T_max_ (°C)	T_min_ (°C)	The hours that temperature >32 °C per day (h d^−1^)
2010	*Simian 3*	Elevated	34.6	36.5	31.6	43.0	30.2	17
		Ambient	31.7	33.7	28.4	40.1	26.7	9
	*Siza 3*	Elevated	34.7	36.6	31.7	43.1	30.3	17
		Ambient	31.4	33.4	28.1	39.7	26.5	9
2011	*Simian 3*	Elevated	32.1	33.7	29.8	38.6	27.8	9
		Ambient	29.1	30.6	26.7	35.2	25.5	5
	*Siza 3*	Elevated	32.2	33.7	30.0	38.9	27.9	9
		Ambient	29.1	30.6	26.7	35.2	25.5	5
2012	*Simian 3*	Elevated	32.4	33.8	29.9	38.2	27.4	12
		Ambient	29.9	31.3	27.3	35.9	25.4	7
	*Siza 3*	Elevated	32.0	33.3	29.8	37.8	27.4	11
		Ambient	28.9	30.3	26.5	35.0	24.6	6

MDT, Mean daily temperature; T_d_, mean daytime air temperature; T_n_, mean nighttime air temperature; T_max_, mean daily maximum air temperature; T_min_, mean daily minimum air temperature.

**Table 2:** Relative humidity in cotton field within 24 hours, at 15 DPA in 2010.

Cultivar	Time (hh:mm)	Temperature regime	Relative humidity (%)		
			FB_2_	FB_6_	FB_10_
*Simian 3*	2:00	Elevated	90 a	89 a	89 a
		Ambient	86 a	86 a	86 a
	8:00	Elevated	78 a	77 a	73 a
		Ambient	75 a	74 a	72 a
	14:00	Elevated	60 a	64 a	58 a
		Ambient	56 a	56 a	56 a
	20:00	Elevated	89 a	89 a	87 a
		Ambient	85 a	84 a	83 a
*Siza 3*	2:00	Elevated	87 a	87 a	86 a
		Ambient	87 a	89 a	88 a
	8:00	Elevated	73 a	73 a	72 a
		Ambient	74 a	75 a	75 a
	14:00	Elevated	57 a	56 a	53 a
		Ambient	56 a	52 a	50 a
	20:00	Elevated	82 a	83 a	81 a
		Ambient	85 a	85 a	85 a

FB_2_, FB_6_, FB_10_ represent the 2^nd^, 6^th^, 10^th^ fruiting branch, respectively. Data followed by different letters between two treatment at one time on the same FB represent statistically significant differences at p ≤ 0.05 level based on ANOVA. Data were recorded at the 15 DPA based on FB_6_, 1^st^ node.

### Effect of temperature elevation on fiber elongation

**Figure 4:**
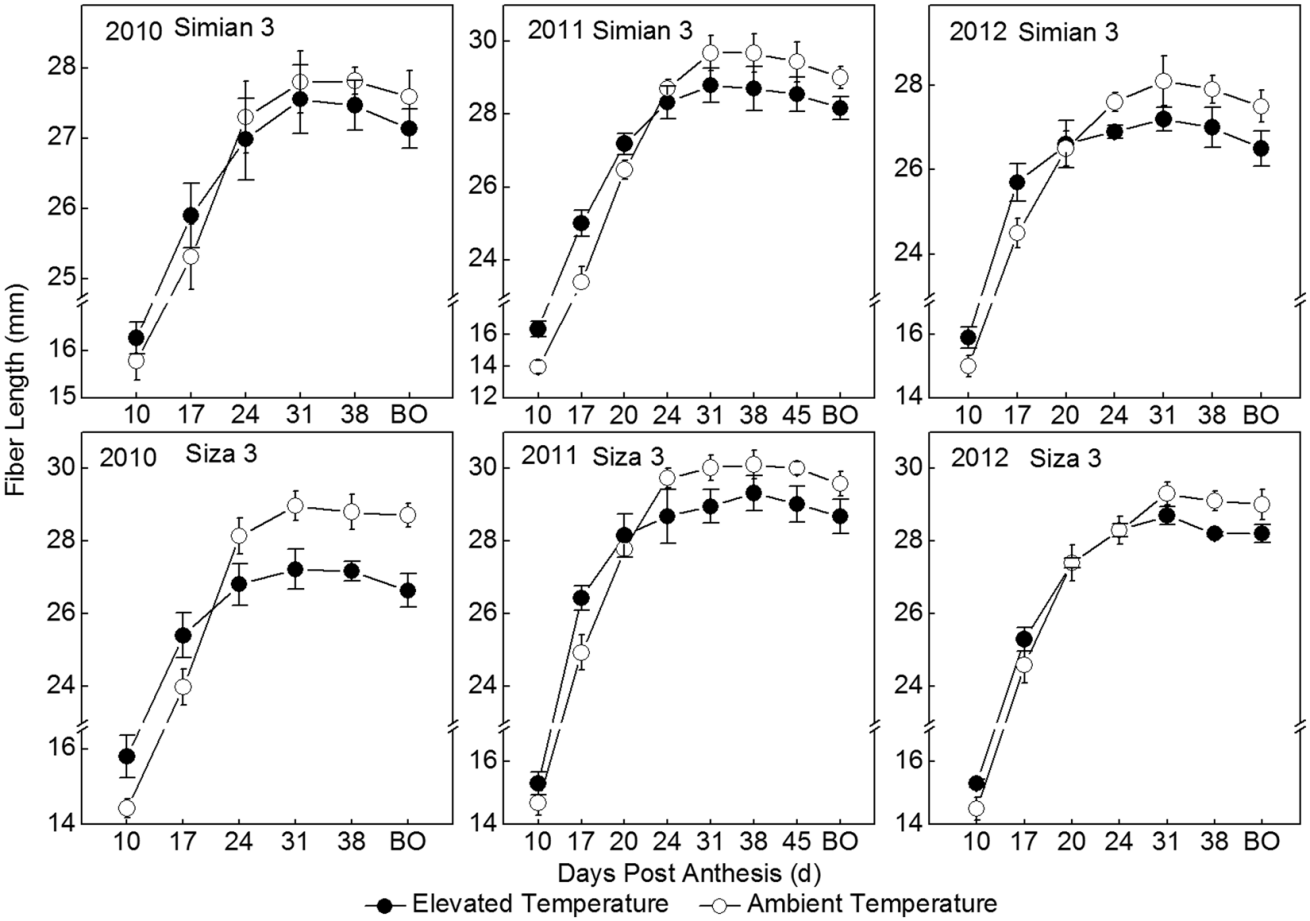
Dynamic changes of cotton fiber length with days post anthesis (DPA) in two temperature regions. BO indicates boll opening. Cotton fibers (bolls) in test were sampled at the first node of the 6^th^ main-stem fruiting branch. Cotton bolls were matured (opened) at 41 and 42 DPA under both the elevated temperature treatment and the control, respectively, in 2010. The corresponding DPA were 46 and 49 DPA in 2011, and were 43 and 46 DPA in 2012. Each data point before 31 DPA represents the mean ± SE of 20 biological repeats; when after 31 DPA, it represents the mean ± SE of 10 biological represent.

Fiber length increased remarkably before 24 DPA, and slowed down after that, and became constant until boll opening (Fig. 4). It was obvious that the fibers grown under elevated temperature were significantly shorter than that of control (Table 3). Final fiber length of Simian 3 and Siza 3 under elevated temperature was 1.8–4.0% and 2.8–7.3% shorter than control, respectively (Table 4). Actually before 20 DPA, fibers grown under elevated temperature were momentarily longer than control; however their elongation rate slowed down in advance which eventually resulted in shorter length (Fig. 4). On the whole, a turning point existed around 20 DPA, after which the response of fiber elongation to temperature elevation was changed (Table 4, Fig. 4).

**Table 3:** Eigen-values of cotton fiber elongation with cotton cultivars of Simian 3 and Siza 3 under the elevated and ambient temperature regimes from 2010 to 2012.

Cultivars	Year	Temperature regime	R^2^	FED	FRED		V_max_		Len_m_ (mm)	Len_obs_ (mm)
					(d)	95% Conf.	(mm·d^−1^)	95% Conf.		
*Simian 3*	2010	Elevated	0.997**	22	7	0.44	2.43	0.14	27.3	27.1 a
		Ambient	0.999**	27	9	0.31	2.04	0.06	27.7	27.6 a
		CTE (%)		−18.5	−22.2		19.1		−1.4	−1.8
	2011	Elevated	0.975**	26	9	0.17	2.10	0.04	28.5	28.5 a
		Ambient	0.981**	28	10	0.07	1.87	0.01	29.5	29.1 a
		CTE (%)		−7.1	−10.0		12.3		−3.5	−2.1
	2012	Elevated	0.988**	21	7	0.03	2.52	0.01	26.8	26.4 b
		Ambient	0.981**	25	8	0.09	2.18	0.02	27.7	27.5 a
		CTE (%)		−16.0	−12.5		15.6		−3.3	−4.0
CV across years (%)		Elevated			13.0		9.37		3.1	3.9
		Ambient			11.0		7.44		3.6	3.2
*Siza 3*	2010	Elevated	0.996**	21	8	0.15	2.35	0.05	27.0	26.6 b
		Ambient	0.998**	30	11	0.19	1.69	0.03	29.0	28.7 a
		CTE (%)		−30.0	−27.3		39.1		−6.8	−7.3
	2011	Elevated	0.988**	24	8	0.13	2.38	0.04	29.0	28.7 b
		Ambient	0.983**	28	9	0.12	2.12	0.02	29.9	29.8 a
		CTE (%)		−14.3	−11.1		12.3		−2.8	−3.7
	2012	Elevated	0.987**	27	8	0.13	2.34	0.04	28.3	28.2 b
		Ambient	0.982**	32	9	0.15	2.12	0.03	29.0	29.0 a
		CTE (%)		−15.6	−11.1		10.4		−2.6	−2.8
CV across years (%)		Elevated			3.1		1.0		3.7	3.9
		Ambient			12.7		12.7		1.7	2.0

FRED, fiber rapid−elongation duration; V_max_, the maximum fiber elongation rate; 95% Conf., 95% confidence interval; Len_m_, theoretical maximum fiber length; Len_obs_, observed fiber length; CTE, Coefficient of temperature elevation; CV, Coefficient of variation; **significant difference at 0.05 probability level (n = 6, R^2^_0.05_ = 0.6584, R^2^_0.01_ = 0.8413; n = 9, R^2^_0.05_ = 0.4440, R^2^_0.01_ = 0.6354; n = 10, R^2^_0.05_ = 0.3994, R^2^_0.01_ = 0.5885). Values followed by a different letter within the same column are significantly different at 0.05 probability levels.

**Table 4:** Coefficients of temperature elevation (CTE, %) of cotton fiber length during fiber elongation, from 2010 to 2012.

DPA (d)	Coefficients of temperature elevation (CTE, %)					
	Simian 3			Siza 3		
	2010	2011	2012	2010	2011	2012
5	nd	20.45*	23.58**	nd	14.63**	17.29**
10	2.99**	17.12**	6.30**	9.47**	4.16**	5.25**
15	nd	9.77**	5.39**	nd	6.01**	6.35**
17	2.31**	8.92**	4.71*	5.91**	6.82**	2.67*
20	nd	2.72**	0.39	nd	1.38	0.07
24	−1.13	−1.30	−2.57**	−4.72**	−3.51**	0.02
31	−0.85	−3.00**	−3.53*	−6.03**	−3.53**	−2.13*
38	−1.27	−3.25**	−3.20	−5.66**	−1.28	−2.86**
45	nd	−3.00	nd	nd	−1.97*	nd
Boll opening	−1.81	−2.06	−4.00*	−7.32**	−3.69**	−2.76**

CTE, coefficient of temperature elevation was calculated by “(Temperature elevation − Control)/Control × 100%”; * and ** represent significant difference at 0.01 and 0.05 probability levels, respectively, between two temperature regions, respectively.

Logistic function can be used for simulating the dynamic change of fiber length according to its growth curve^34^. The methods for calculating eigen-values were listed in Methods/Statistical analysis. By analyzing the coefficients of temperature elevation (CTE), both the maximum fiber elongation rate (V_max_) and fiber rapid-elongation duration (FRED) contributed significantly to the final fiber length (Table 3). The FRED under elevated temperature was shortened by 1–3 and 1–4 days for Simian 3 and Siza 3, respectively. This temperature elevation made cotton fibers premature and terminated elongation in advance. Compared to FRED, the response of V_max_ to temperature elevation was relatively more obvious (see CTE in Table 3). The V_max_ was 2.10–2.52 mm·d^−1^ in the thermo treatment, but only 1.69–2.18 mm·d^−1^ in the control. As a result, both the theoretical maximum length of fiber (Len_m_) and the observed fiber length (Len_obs_) under thermo treatment were reduced. The inter-annual variations of Len_m_ and Len_obs_ between the two temperature-regimes were almost the same for Simian 3 (see CV in Table 3). However, for Siza 3, the CV under elevated temperature was much higher than that of control, which presented an evidence of genotypic difference in thermo stabilities.

It was noticeable that Len_obs_ had significantly negative correlations with all the temperature indices measured in the current study, including the daily hours of temperature >32 °C (Table 5). As T_n_ and the hours of temperature >32 °C per day were significantly correlated with all the eigenvalues, we speculated that the T_n_ and the hours of excessively high temperature in daytime together played dominant roles in cotton fiber elongation when responding to high temperature stress.

**Table 5:** Correlation coefficients for temperature indices and eigen-values of cotton fiber elongation with cotton cultivars of Simian 3 and Siza 3 under the elevated and ambient temperature regimes from 2010 to 2012.

Temperature indices	Correlation coefficient (r)		
	V_max_	FRED	Len_obs_
MDT	0.547	−0.53	−0.730**
T_d_	0.472	−0.461	−0.712**
T_n_	0.650*	−0.611*	−0.688*
T_max_	0.319	−0.321	−0.650*
T_min_	0.555	−0.527	−0.633*
Hours T > 32 °C/d	0.592*	−0.591*	−0.788**

MDT, mean daily temperature; T_d_, mean daytime air temperature; T_n_, mean nighttime air temperature; T_max_, mean daily maximum air temperature; T_min_, mean daily minimum air temperature; FRED, fiber rapid-elongation duration; V_max_, the maximum fiber elongation rate; Len_obs_, observed fiber length; * and ** mean significant difference at 0.01 and 0.05 probability level, respectively; n = 12, *R*_0.05_ = 0.576, *R*_0.01_ = 0.707.

### Enzymes activities and genes expression quantities in fiber responding to excessively high temperature

**Figure 5:**
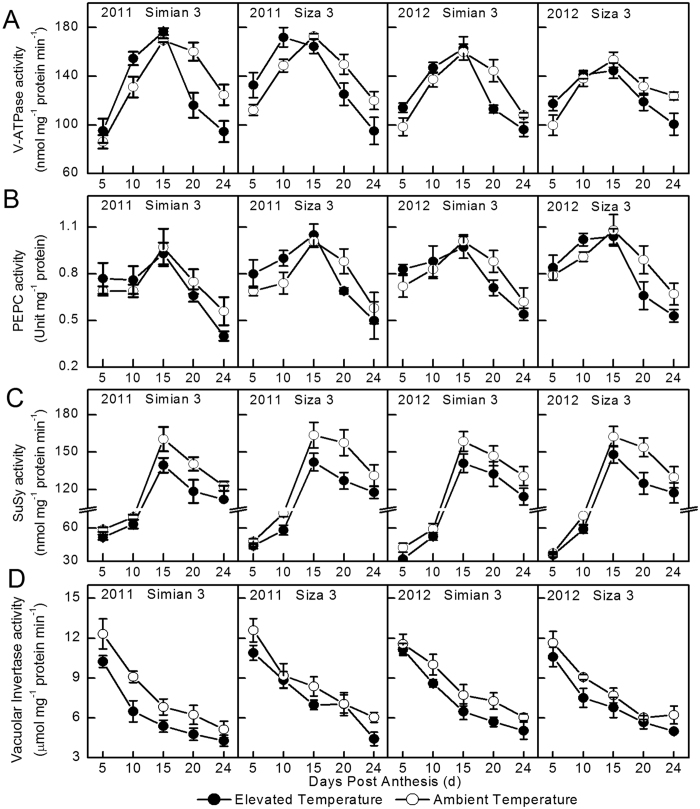
Dynamic changes of V-ATPase (**A**), PEPC (**B**), SuSy (**C**) and VINV activities (**D**) in cotton fiber in two temperature regions. Each data point represents the mean ± SE of three biological repeats.

The activity variations of V-ATPase, PEPC and SuSy across DPA followed single peak curve, where peak values emerged around 15 DPA (Fig. 5A–C). Before 15 DPA, the activities of V-ATPase and PEPC under elevated temperature were higher than those under control, however, this tendency reversed after 15 DPA (Fig. 5A,B). Regarding to SuSy and VINV, their activities under elevated temperature were consistently lower than that of control (Fig. 5C,D).

**Figure 6:**
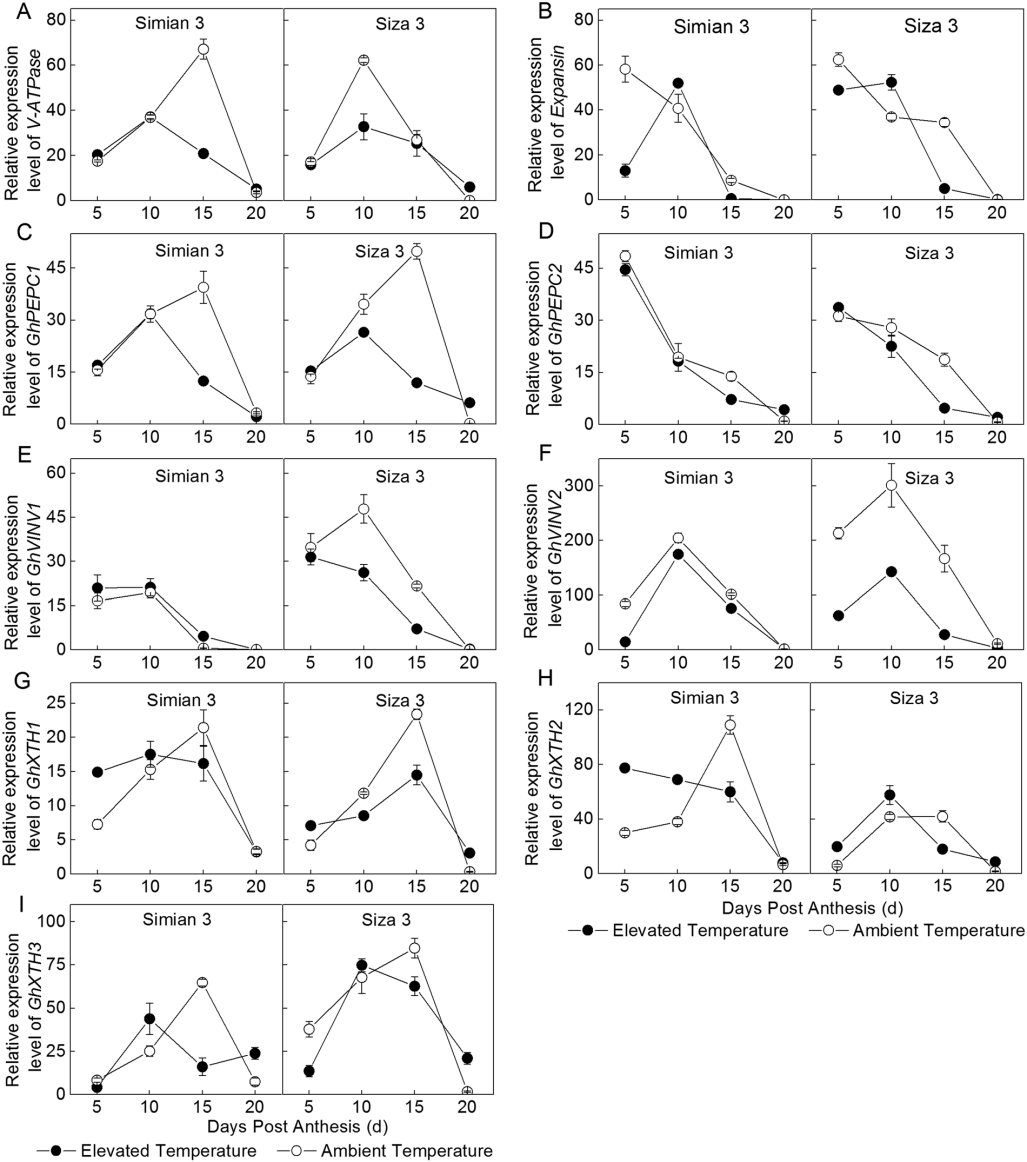
Relative expression quantities of key genes engaged in cotton fiber development in two temperature regimes, in 2012. *V-ATPase* (**A**), *Expansin* (**B**), *GhPEPC1* (**C**), *GhPEPC2* (**D**), *GhVINV1* (**E**), *GhVINV2* (**F**), *GhXTH1* (**G**), *GhXTH2* (**H**), *GhXTH3* (**I**). Each data point represents the mean ± SE of three biological repeats.

The expression quantity of *V-ATPase* increased before 15 DPA and then decreased noticeably after that, especially the fibers grown in elevated temperature region (Fig. 6A). The Most peak values of gene expression quantity under elevated temperature were emerged at 15 DPA and 10 DPA for Simian 3 and Siza 3, respectively, and most of those were significantly higher than control (*P* < 0.05). *V-ATPase* was a potential down-regulated gene when suffering thermo stress. The expression level of *Expansin* under thermo treatment was down-regulated at 5 DPA, peaked at 10 DPA, then decreased rapidly (Fig. 6B), which differed from control that decreased continuously from beginning to the end. Figure 4(C) indicated that the gene expression quantity of *GhPEPC1* across DPA followed single peak curve and the expression quantity under elevated temperature was significantly lower than that of control. Moreover, the peak value of the former emerged 5 days ahead of the later. The *GhPEPC2* expression level kept declining from 5 to 20 DPA, and the gap between elevated and ambient temperature regions was little at 5 DPA, but significant at 15 DPA (Fig. 6D). The expression quantity of VINV genes at 10 DPA was higher and then declined rapidly (Fig. 6E,F). Meanwhile, the expression levels of *GhVINVs* (*GhVINV1*, *GhVINV2*) were evidently down-regulated for Siza 3 but not for Simian 3, when fibers grown in elevated temperature region. As shown in Fig. 6(G–I), *GhXTH1*, *GhXTH2* and *GhXTH3* were the most highly expressed at 10 and 15 DPA when under elevated temperature and control, respectively, while, the peak values of control were much higher.

**Figure 7:**
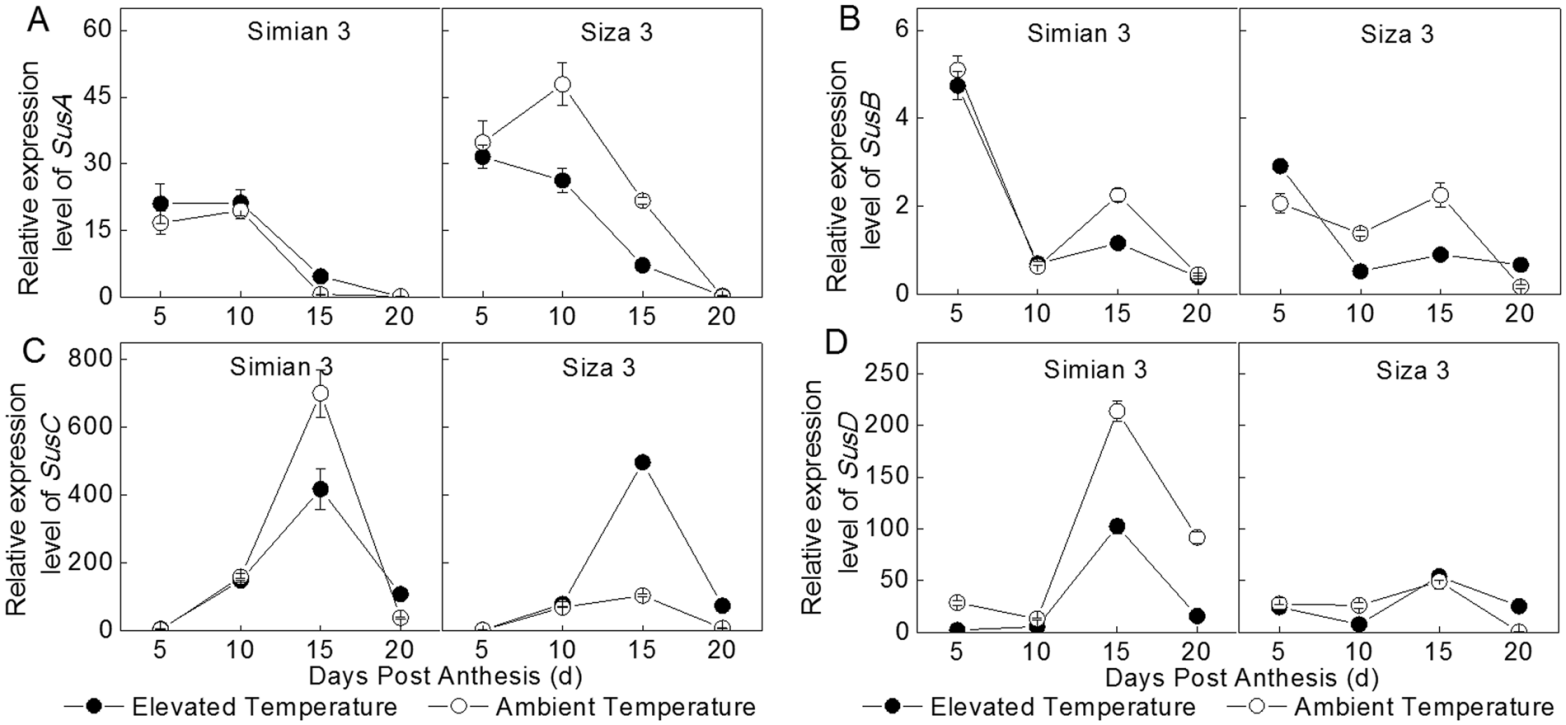
Relative expression quantities of key genes engaged in cotton fiber development in two temperature regimes, in 2012. *SusA* (**A**), *SusB* (**B**), *SusC* (**C**), *SusD* (**D**). Each data point represents the mean ± SE of three biological repeats.

Figure 7 exhibited gene expressed magnitude of SuSy isozymes. *SusC* and *SusD* expressed high, while *SusB* expressed extremely low. Regardless of *SusB*, the peak values of *SusA* emerged at 10 and 15 DPA for *SusC* and *SusD*, respectively. The *SusA* expression levels from two temperature regimes had significant difference for Siza 3, but had little difference for Simian 3. The regularity reversed on *SusD*. Furthermore, *SusC* was up-regulated for Simian 3, but down-regulated for Siza 3 under elevated temperature.

## Discussion

The upper limit of the tolerable temperature for cotton fiber growth is 32/28 °C (T_d_/T_n_)^32, 33^. Therefore, the air temperature >32 °C caused by global warming would certainly pose a challenge to cotton production in the future^35^. In the current study, the T_d_/T_n_ in elevated temperature region during fiber elongation was (33.3–36.6)/(29.8–31.7) °C (Table 1), which has exceeded the upper limit. This temperature stress has accelerated fiber elongation process (Fig. 4), which potentially caused by FRED shortening (Table 3). Although this negative factor was counteracted by a greater V_max_ to some extent^36^, it was far from enough to offset the suppression on fiber length. It was worthy to mention that, compared with T_d_, T_n_ had a greater influence on fiber elongation (Table 5), which means T_n_ could be deemed as an ideal index for forecasting the degree of high temperature stress would happen to cotton fiber length in future.

It was obvious that the effect of elevated temperature on fiber elongation was different in the early and late elongation phases (Fig. 4). Its sensitivity was gradually decreasing along with fiber growth (Fig. 4, Table 4). Two explainations could be proposed. Firstly, the oxygenic atmosphere in nascent organs is commonly higher under high temperature stress^37^, which was possibly because of the large demand for cell metabolism, and was helpful to cope with suboptimum conditions^37^. Secondly, in the current study, the related transporters^38^ and some key enzymes showed reversed activities between the early and late elongation phase when under temperature stress (Fig. 5A,B), which phenomenon was similar to the resposne of fiber length.

As an essential enzyme involved in malate bio-synthesis, PEPC is implicated in plant cell expansion through osmoregulation^18^. In the present study, fiber PEPC activity remained higher during the rapid elongation phase but stepped down after 15 DPA (Fig. 5B), especially under high temperature stress. Acturally, high PEPC activity might also required for membrane lipids synthesis, which is a necessary process for membrane enlargement and fiber elongation^16^. This might be another cause for the sluggish in fiber development after 15 DPA (Fig. 4). Under elevated temperature, the appearance of the peak transcript level of *V-ATPase* and *GhPEPC1* have been advanced from 15 DPA to 10 DPA (Fig. 6A,C), resulted in higher enzyme activities during early elongation phase (5–15 DPA) (Fig. 5A,B). This was beneficial for maintaining great amount of malate, and the homeostasis between ions and metabolites^21, 39^, and contributed to get higher fiber V_max_ (Table 3). However, when the secondary cell wall construction phase came (after 15 DPA), the fiber expansion-related genes, as well as the V-ATPase and PEPC activities were consistently down regulated^22, 40^. These physiological variation would negatively affect the osmotically active solutes accumulation^18^, especially for the elevated temperature treatment, and resulted in shorter FRED^18, 41^, at last. Besides, the persistent decrease of SuSy activity (Fig. 5C), mainly caused by *SusA*, *B* and *D* suppression (Fig. 7A,B and D), has additionally limited cell turgor establishment^23^. As a result, cotton fiber under elevated temperature could not persistently maintain high cell turgor for a long time, compared to control, which was embodied in FRED in the current study (Table 3).

Cotton fiber cell wall enlargement is mainly regulated by the xyloglucan endotransglycosylase/hydrolases (XTH)^42^. Under elevated temperature, *GhXTH1* and 2 were up-regulated from 5 to 10 DPA, and only at 10 DPA for *GhXTH3* (Fig. 6G–I). These three genes were conducive to increase XTH activity and could assist fiber cell expansion^28, 43^. However, these three genes were consistently down-regulated after 15 DPA, which promoted xyloglucan over-accumulation, and limited cell enlargement^44^, indirectly. We suggested that fiber *GhXTH1* and 2 were sensitive genes responding to elevated temperature during fiber’s early elongation phase. In addition, another down-regulated gene, *Expansin* (Fig. 6B), which is responseible for expansin protein synthesis, also assisted obstructing fiber enlargement.

Fiber properties, especially fiber length, are primarily dependent on genetics^13^. However, fiber properties among similar cultivars or even the same variety could be significantly changed by temperature variation^30^. Two cotton cultivars used in the present study, Simian 3 (conventional non-hybrid cotton) and Siza 3 (hybrid cotton), were bred and planted widely in the Yangtze River Valley, China, in different years. Both cultivars were well adapted to the environment and gained good fiber quality in each planted year. According to the ANOVA in Table 3, the fiber length of Siza 3 grown under elevated temperature was consistently shorter, but Simian 3 was little affected (Table 3). This result suggests that Siza 3, compared with Simian 3, was more sensitive to high temperature stress which means it would perform worse in the warming future.

Overall, considering the sparse information on how high temperature in future would affect fiber properties, this study advanced the understanding. The activities of V-ATPase, PEPC, SuSy and VINV, and the expression levels of *V-ATPase*, *Expansin*, *GhPEPCs*, *GhVINVs*, *GhXTHs*, *SusA* and *SusD*, together regulated fiber FRED, which accordingly decreased fiber length. However, it is still unclear which gene or metabolic process plays the dominant role. Nonetheless, using two cultivars might be not enough to fully prove this issue. Therefore, more detailed research we are focusing on.

## Methods

### Plant materials and experimental design

Field experiments were conducted at Pailou Experimental Base (118°50′E, 32°02′N), Nanjing Agricultural University, Nanjing, China, during 2010 to 2012 cotton growing seasons to study the effects of elevated temperatures on fiber elongation and fiber length of upland cotton (*Gossypium hirsutum* L.). Two cotton cultivars Simian 3 and Siza 3 were used in these experiments. Simian 3, conventional non-hybrid cotton, was examined and approved by Jiangsu province and China National Variety Examination and Approval Committee in 1993 and 1995, respectively, and was widely planted in the Yangtze River Valley cotton belt in 1990s. Siza 3, hybrid cotton, was examined and approved by Jiangsu province and China National Variety Examination and Approval Committee in 2005 and 2008, respectively, and has been widely planted in the Yangtze River Valley cotton belt since start of 21^st^ century. The growth period was about 130–135 days for Simian 3 and about 130 days for Siza 3, and the lint yield of Siza 3 was 8.0–15.7% higher than Simian 3^45^. In short, these two cotton cultivars have similar ecological fitness but adapted to different ages.

A Temperature Control System (OTC, Southeast Co. Ltd, Ningbo, China) was used in the field experiments for maintenance of microclimate temperature effects (Figs 1 and 2). There was no temperature treatment applied before the appearance of white flower at the first position of the 6^th^ main-stem fruiting branch. Experimental treatments during flowering and boll formation stages were (a) ambient temperature and (b) elevated temperature, which was 2–3.5 °C warmer than ambient temperature across flowering and boll formation stage (Fig. 3). Temperature data during fiber elongation in 3 experimental years has been listed in Table 1. Temperature elevation was achieved by the Temperature Control System (Fig. 1). Surrounding ambient air was elevated by a compressor (5.5 kW, CC-107, Ningbo Southeast Co., China) and blown into cotton field by an air blower (350 W, AH9, Ningbo Southeast Co., China) connected with a major duct and 36 sub-ducts (Fig. 2). On the sub-ducts, small holes were drilled with uneven distance to make sure that very similar volume of air released to each area. Six temperature and humidity sensors are evenly placed to record the real-time data at a 20-min interval in both treatment and reference field (Fig. 3). Both treatment and reference field have no roof and are surrounded with 1-meter-high thick transparent plastic.

Cotton seeds were planted in a nursery bed on 25^th^ of April and were transplanted into bottomless pots, half buried in soil, on 15^th^ of May when the seedlings had third true leaves. Soil at the experimental site was Typic Dystrudept, and its organic matter and nitrogen, phosphorus and potassium contents are listed in Table 6. Other environmental factors were kept constant between two fields, e.g. an adequate supply of water and nutrients, and precise insect control.

**Table 6:** Characteristics of soil fertility from 2010 to 2012.

Year	Organic matter content (g kg^−1^)	Total N content (g kg^−1^)	Mineral N content (NH_4_^+^ and NO_3_^−^, mg kg^−1^)	Olsen P content (mg kg^−1^)	Exchangeable K content (NH_4_OAC-K, mg kg^−1^)
2010	16.5	1.0	50.5	16.8	95.5
2011	18.3	1.2	74.2	18.4	101.7
2012	17.8	1.1	76.4	17.7	111.4

### Sampling and processing

White cotton flowers at the first node of the 6^th^ main-stem fruiting branch of all plants were tagged with small plastic tags, labeling the flowering dates. 6–8 tagged normal bolls were collected every 7 days from 10 DPA until boll opening. In order to ensure the accuracy of results, 3 additional sampling points were added at 5 DPA, 15 DPA and 20 DPA in 2011 and 2012. Cotton bolls were harvested at 9:00–10:00 am local time. Four locules from four bolls of each sampling point were used for measuring fiber length, other locules and bolls were used to measure fiber development, enzymes activities and genes expressions. Samples during 10–24 DPA for enzymatic and genetic measurements were excised from the bolls with a scalpel and the bolls were put on ice during the operation. Subsequently, both target fibers and seed cotton at 5 DPA were immediately placed in liquid nitrogen and stored at −80 °C for subsequent enzymatic measurement. 10 normal size bolls per experimental plot were harvested for final fiber length analyzing when tagged bolls opened.

### Fiber length measurement

Because of the fragility and high soluble sugar content, young cotton fibers, before 31 DPA, were oven dried, and their lengths (mm) were measured by water washing method^8, 11^. Four locules from different four bolls were placed in boiling 0.1 HCl for 3–5 min till the seeds were separated from each other. Then, five seeds were selected randomly from each locule. Each seed was placed on a convex surface of a watch glass. Fibers were streamed out with a jet of water, and their length was measured with a vernier caliper from the attachment point on epidermis to the edge where most fibers terminate. Old cotton fibers, after 31 DPA, cannot be streamed straightly because of crimp, therefore, their length was measured by a Y-146 photoelectric stapler (Taicang Electron Apparatus Co., Ltd., China), after oven dried at 60 °C for 0.5 h, then at 40 °C for 48 h. Before measurement, lint samples after oven drying were placed in a testing room with constant temperature and humidity [(20 ± 2), (65 ± 2)%, relative humidity] for 48 h for stabilization. Cotton fibers of 31 DPA were measured by both water washing and photoelectric stapler methods for reducing the systematic errors, in which the latter method shall prevail.

An additional large sample test on matured cotton fiber length (boll opened, BO) was performed by an USTER HVI MF100 cotton fiber quality measurement system (Uster Technologies Co., Ltd., Switzerland). Compared to former two methods, the HVI results were gained after lint harvest instead of sampling, with the purpose of reinforcing data credibility. It is worth mentioning that cotton bolls at the first node of the 6^th^ main-stem fruiting branch in 2010 were matured and boll opened at 41 DPA under the elevated temperature treatment, and at 42 DPA under control. The time points were 46 and 49 DPA in 2011, and were 43 and 46 DPA in 2012. In addition, there was little difference existed between two cultivars on their boll periods when under the same condition.

### Enzymatic analyses

Plasma membrane (PM) extraction method and activity assay method were according to Smart *et al*.^22^. V-ATPase activity, expressed as micromoles per minute per milligram of protein, was assayed as the liberation of Pi from either ATP or PPi and was detected colorimetrically, andcalculated as the difference in Pi released assayed in the presence of Cl^−^ or NO_3_^−^ ions^22^. PEPC activity was assayed spectrophotometrically at 340 nm at 24 °C. The reaction was enzymatically coupled to malate dehydrogenase (EC 1.1.1.37), and the rate of NADH oxidation was monitored^22^.

SuSy and VINV extraction and assay were according to King *et al*.^46^. SuSy activity was assayed by measuring the cleavage of sucrose^46^. VINV activity was measured by incubation of 100 μl of extract with 1 M sucrose in 200 mM acetic acid - NaOH (pH 5.0), in a total volume of 2.5 ml, and glucose content was determined with a spectrophotometer at 540 nm^46^.

### Gene expression by quantitative real-time PCR (qRT-PCR)

Total RNA from cotton fiber was extracted using modified hot borate method according to Wu and Liu^47^. Total RNA was reverse transcribed using TaKaRa one step RT-PCR kit (TaKaRa, Dalian, China). Premier 6.0 and Beacon designer 7.8 were used for PCR primer design and synthesis. Reactions were conducted under the following step in supplementary table. The qRT-PCR analysis was performed using the CFX384 multiplex real-time fluorescent quantitative PCR instrument (Bio-Rad, USA) according to the manufacturer’s protocol. A 40-cycle two-step amplification protocol (10 s at 95 °C, 25 s at 64 °C) was used for all measurements. All primers used for quantitative RT-PCR are listed in supplementary table. PCR amplification of 18 S rRNA was performed for normalization between treated and control samples.

### Temperature data

Temperature data was recorded every 20 min by the temperature control device (Temperature Control System). The mean daily air temperature (MDT), mean daytime air temperature (T_d_), mean nighttime air temperature (T_n_), mean daily maximum air temperature (T_max_), mean daily minimum air temperature (T_min_) and the hours of temperature >32 °C per day during fiber elongation were exported from the temperature control device.

### Statistical analysis

Data were subjected to an analysis of variance with SPSS statistic package Version 17.0 and the difference between mean values greater than LSD (*P* ≤ 0.05) was determined as significant. CTE, coefficient of temperature elevation = (elevated temperature − control)/control × 100%, was calculated for understanding the effects of elevated temperature on fiber length and comparing the variance with control. The coefficient of variation (CV%) was calculated as the ratio of the standard deviation to the mean.

### Eigen-values of cotton fiber elongation analysis

The formation of fiber length can be described by logistic model^34, 48^. In equation (1), Len represents fiber length, Len_m_ represents the theoretical maximum of fiber length; in equations (2), (3) and (4), DPA_1_, DPA_2_ and V_max_ stand for the start DPA of fiber elongation, the termination DPA of fiber elongation and the maximum fiber elongation rate, respectively. In the equations below, “a” and “b” are parameters. Fiber rapid-elongation duration (FRED) was calculated as DPA_2_ - DPA_1_.

1 $$Len=\frac{Le{n}_{m}}{1+a{e}^{b\times DPA}}$$

2 $$DP{A}_{1}=\frac{1}{b}ln\frac{2+{3}^{\frac{1}{2}}}{a}$$

3 $$DP{A}_{2}=\frac{1}{b}ln\frac{2-{3}^{\frac{1}{2}}}{a}$$

4 $${V}_{max}=-\frac{b\times Le{n}_{m}}{4}$$

## Electronic supplementary material

Supplementary Information
