# Supplementary material for: Simulative Global Warming Negatively Affects Cotton Fiber Length through Shortening Fiber Rapid Elongation Duration
Source: Sci Rep. 2017 Aug 23;7:9264. doi: 10.1038/s41598-017-09545-y (PMC5569071; doi:10.1038/s41598-017-09545-y)
Supplement: Supplementary file 1 — Supplementary Information [file 41598_2017_9545_MOESM1_ESM.doc]

**Simulative Global Warming** **Negatively Affects Cotton Fiber Length through Shortening Fiber Rapid Elongation Duration**

**Authors:** Yan-Jiao Dai^†^, Jia-Shuo Yang^†^, Wei Hu, Rizwan Zahoor, Binglin Chen, Wenqing Zhao, Yali Meng, Zhiguo Zhou[[1]](#footnote-2)*

**Affiliation:** Key Laboratory of Crop Physiology & Ecology, Department of Agronomy, College of Agriculture, Nanjing Agricultural University, Nanjing, Jiangsu Province, China

^†^ Yan-Jiao Dai and Jia-Shuo Yang contributed equally to this work

*Corresponding author: Zhiguo Zhou

E-mail: giscott@njau.edu.cn

Tel/Fax: 86-25-84396813

**Correspondence address:** Department of Agronomy, College of Agriculture, Nanjing Agricultural University, Nanjing, Jiangsu Province, China

**E-mail address:** Yanjiao Dai: 2010201032@njau.edu.cn

Jiashuo Yang: 2013201029@njau.edu.cn

Wei Hu: 2012201026@njau.edu.cn

Rizwan Zahoor: rizwaagr@gmail.com

Binglin Chen: blchen@njau.edu.cn

Wenqing Zhao: zhaowenqing@njau.edu.cn

Yali Meng: mengyl@njau.edu.cn

**Number of words:** 2,515 (only including Introduction, Results and Discussion)

**Number of figures:** 7

**Number of tables:** 6

**Supplementary table:** **Quantitative real-time PCR Primers and conditions in the experiment.**

| **Gene** | **Genbank**  **Accession** | **Primer Sequence**  **(5'-3')** | **Size**  **(bp)** | **Annealing**  **(°C)** |
| --- | --- | --- | --- | --- |
| *V-ATPase* | L03186.1 | CGTTGCTGATGGAATGGCTGGT | 99 | 63 |
|  |  | GCAGAGTCTCCTTCCAACCGA |  |  |
| *Expansin* | JN255197.1 | CTCCGGCAGCCCTTCCAT | 58 | 63 |
|  |  | GCACCAGCCACCATTGTCATTT |  |  |
| *GhPEPC1* | AF008939.1 | GGAAACCCCAGGGTAACTCCT | 125 | 63 |
|  |  | CTGCAACGCCACATTGATAACTCA |  |  |
| *GhPEPC2* | AF008940.1 | CCTGCTGATGAACTTGTCAAACTG | 83 | 63 |
|  |  | CCCTTCATGGTCAAGATGAGG |  |  |
| *GhVINV1* | FJ915120.1 | CCATGCTCCCTTGCTACACCA | 169 | 63 |
|  |  | CGACCCTGCCTCCGTTACACTA |  |  |
| *GhVINV2* | FJ864677.1 | GGATCTGTTGTGCCCCTTGACA | 143 | 63 |
|  |  | GGTCCGTAAGTGCTCCTATCA |  |  |
| *GhXTH1* | HM749062.1 | CCGGTTCCAGGGCCAGTAAA | 149 | 63 |
|  |  | CGGGACTTGTCGGTGCAGTAAT |  |  |
| *GhXTH2* | HM749061.1 | CAGTTCCAGGGCCAGCAAATT | 147 | 63 |
|  |  | GAGACTTGTCGGTGCAGTAATCGT |  |  |
| *GhXTH3* | HM749060.1 | CGGACGTGTCAGCATGAAGATC | 133 | 63 |
|  |  | GCCGCTACGGTTTCCCAAGA |  |  |
| *SusA* | U73588.2 | CCCACGGATACTTCGCTCAAGAC | 141 | 63 |
|  |  | GAGGGGTGATGTTGAGTCCTTGTT |  |  |
| *SusB* | JN376125.1 | CCGTGCCTTGGAGAACGAGAT | 96 | 63 |
|  |  | GCGTCAGGGAGAAGTCTGGTAAT |  |  |
| *SusC* | JN376126.1 | GTGAGCGTTTGGGTGAATCT | 141 | 63 |
|  |  | GTGCTTGATTTCCGGCCTCT |  |  |
| *SusD* | JN376127.1 | ACGACCACAACAAGCCAATTCTAT | 139 | 63 |
|  |  | CTCCGATCTCCACCGACAACTA |  |  |
| *Gh18S rRNA* | U42827.1 | CAGATACCGTCCTAGTCTCAACCA | 73 | 63 |
|  |  | GGCGGAGTCCTGAAAGCAACAT |  |  |

1. - *To whom correspondence should be addressed.

   Zhiguo Zhou, E-mail address: [giscott@njau.edu.cn](mailto:giscott@njau.edu.cn). [↑](#footnote-ref-2)
